# Choice of Bacterial Growth Medium Alters the Transcriptome and Phenotype of *Salmonella enterica* Serovar Typhimurium

**DOI:** 10.1371/journal.pone.0063912

**Published:** 2013-05-21

**Authors:** Jessica M. A. Blair, Grace E. Richmond, Andrew M. Bailey, Al Ivens, Laura J. V. Piddock

**Affiliations:** 1 Antimicrobials Research Group, School of Immunity and Infection and Institute of Microbiology and Infection, University of Birmingham, Edgbaston, Birmingham, United Kingdom; 2 Centre for Immunity, Infection and Evolution, University of Edinburgh Ashworth Labs, King’s Buildings, Edinburgh, United Kingdom; University of Glasgow, United Kingdom

## Abstract

The type of bacterial culture medium is an important consideration during design of any experimental protocol. The aim of this study was to understand the impact of medium choice on bacterial gene expression and physiology by comparing the transcriptome of Salmonella enterica SL1344 after growth in the widely used LB broth or the rationally designed MOPS minimal medium. Transcriptomics showed that after growth in MOPS minimal media, compared to LB, there was increased expression of 42 genes involved in amino acid synthesis and 23 genes coding for ABC transporters. Seven flagellar genes had decreased expression after growth in MOPS minimal medium and this correlated with a decreased motility. In both MOPS minimal medium and MEM expression of genes from SPI-2 was increased and the adhesion of *S*. Typhimurium to intestinal epithelial cells was higher compared to the levels after growth in LB. However, SL1344 invasion was not significantly altered by growth in either MOPs minimal media or MEM. Expression of SPI-2 was also measured using chromosomal GFP reporter fusions followed by flow cytometry which showed, for the first time, that the reduction in SPI-2 transcript after growth in different media related to a reduction in the proportion of the bacterial population expressing SPI-2. These data highlight the profound differences in the global transcriptome after *in vitro* growth in different media and show that choice of medium should be considered carefully during experimental design, particularly when virulence related phenotypes are being measured.

## Introduction

*Salmonella enterica* is the cause of 93.8 million cases of gastroenteritis leading to 155,000 deaths annually worldwide [1]. *Salmonella* is an intracellular pathogen that invades the intestinal epithelia by inducing its own uptake into non-phagocytic enterocytes. This requires a type 3 secretion system (T3SS) encoded by a 40 Kb genomic region called *Salmonella* Pathogenicity Island (SPI) which is required for invasion of host cells [2]. This secretion mechanism injects effector proteins directly into the host cell cytoplasm which initiates rearrangement of the host actin cytoskeleton and destabilisation of host cell tight junctions [3], [4]. A second T3SS, SPI-2, is required for intracellular survival [5], [6], [7], [8], [9]. This T3SS translocates effector proteins across the vacuolar membrane into the host cell allowing bacteria to modulate its external environment and survive within the hostile environment of the host cell. Expression of these T3SSs is bimodal; a proportion of the population express the system, while the remainder do not [10], [11], [12].

Expression of SPI-1 and SPI-2 is regulated by environmental cues that signal to the cell the appropriate time or place for expression. For example SPI-1 is expressed most in conditions that mimic the environment of the small intestine such as slight alkalinity, low oxygen and high osmolarity [13]. In contrast, SPI-2 is induced in conditions similar to the intracellular environment including acidic conditions [14], [15], phosphate starvation [16] and low osmolarity [17], [18]. Therefore, the medium in which bacterial strains are grown alters the expression of these important virulence genes and depending on the research question being investigated researchers are able to choose media which induce the expression of their system of interest. However, commonly the choice of bacterial growth medium in which to perform an experiment is chosen by convention rather than logic without understanding the full consequences of the choice and underestimating the impact of this decision on the outcome of experiments.

The aim of this study was to understand the complete physiological effect of growth in different commonly used bacterial media on the transcriptome of *S*. Typhimurium including genes related to virulence to inform future rational experimental design. In addition, based upon the knowledge that environmental conditions alter expression of Salmonella pathogenicity island genes, the effect of growth medium on the phenotype seen in classical adhesion and invasion studies with *Salmonella enterica* serovar Typhimurium was investigated.

## Materials and Methods

### Strains and Media Used

*S. enterica* serovar Typhimurium SL1344 was used throughout this study [19]. Strains containing chromosomal GFP fusions to *prgH* (SPI1), *ssaG* (SPI2) or *rpsM* (16S control) promoters have been previously described [10]. P22 transduction was used to transfer the GFP fusions into SL1344 giving L1307 (SL1344 ssaG’-gfp+, CmR) and L1309 (SL1344 prgH’-gfp, CmR). Three types of media were compared: LB (Sigma L3022) containing tryptone, yeast extract and NaCl, Minimum essential medium (MEM) (Sigma, M2279) containing inorganic salts, amino acids, vitamins, glucose, NaHCO3, Phenol red and added L-glutamine and NEAA, and MOPs minimal media (Teknova) [20] containing 3-(N-morpholino)propanesulfonic acid, potassium phosphate, glucose and the latter was supplemented with histidine as SL1344 is a histidine auxotroph.

### Determination of Transcriptome

RNA was isolated from strains after growth to mid-logarithmic phase in either LB, defined MOPS minimal medium (Teknova, United States) or MEM (Sigma) at 37°C with shaking at 180 rpm as previously described [21]. Three cultures were grown for each media type, and two RNA preparations made from each culture, giving three biological and two technical replicates of each. RNA was isolated using the Promega SV40 total RNA preparation kit according to the manufacturer’s instructions and ad previously described [22]. The quantity and quality of RNA was determined using an Agilent 2100 Bioanalyser.

Microarray experiments were performed with pan-Salmonella generation IV microarray at the Sanger Genome Campus (Hinxton, United Kingdom) as described previously [22]. Results were analysed using Bioconductor and B values (log odds value of 0, and adjusted *P* value ≤0.05) of ≤0.05 were taken as significant. Microarray data deposited with ArrayExpress, http://www.ebi.ac.uk/arrayexpress/. Accession number E-MTAB-1135. Microarray data were visualised using the Pathway tools software (version 13.0) and the *Salmonella* database (Gemma Langridge, Wellcome Trust Sanger Institute) so that the metabolic genes could be viewed in their respective metabolic pathways enabling themes in the data to be easily identified.

### Quantitative Real-time PCR

Comparative real-time reverse transcription (RT)-PCR was used to validate microarray data and determine the expression of genes of interest in different media types. Sample preparation and real-time RT-PCR was performed according to MIQE guidelines [23]. Technical duplicates of RNA were prepared from biological triplicates of SL1344 grown in LB, MOPs Minimal media and MEM. All samples were additionally treated with TurboDNAse (Ambion) and removal of all DNA was confirmed by a negative PCR result. RNA was quantified by nanodrop (Thermo-scientific). cDNA was synthesised from each RNA preparation using the superscript III cDNA synthesis kit (Invitrogen). Twelve representative genes with a range of functions (biosynthesis, transport and SPI-2 genes) were selected to validate gene expression changes seen in microarrays between SL1344 grown in LB broth and minimal media. Primers were designed with an annealing temperature of 57.3°C using Beacon Designer 4.0 (Premier Biosoft, USA) (Table S1). PCR efficiency validation experiments were carried out using five cDNA standards of different concentrations (10, 1, 0.1, 0.01, 0.001 ng/µl) to determine PCR efficiency for the housekeeping gene 16S and each test gene. qRT-PCR reactions were set up in biological triplicate and technical duplicate in a BIORAD PCR tray using 1 µl neat cDNA for test genes and 1 µl of a 1∶1000 dilution cDNA for 16S. qRT-PCR was carried out in a CFX-96 Real-time machine (BIORAD, UK) and data was analysed using CFX Manager (BIORAD, UK). Expression ratios were calculated using the ΔΔct method, taking into account the primer efficiencies and were normalised to expression of 16S.

### Adhesion and Invasion Assays

SL1344 was grown overnight in either LB broth, MOPS minimal media (Teknova) or MEM (Sigma, M2279) at 37°C, with agitation and washed in PBS before being diluted in MEM supplemented with 1% NEAAs and 1% glutamine. Association and invasion assays with human intestinal epithelial cells (INT-407) were performed as previously described [24], [25], [26]. Briefly, washed monolayers of INT-407 cells were inoculated with 5×10^7^ cfu bacteria and incubated for 2 h at 37°C in 5% CO2. The infected monolayers were then washed and disrupted to allow the number of cfu/mL to be determined by serial dilution onto LB agar. Invasion assays were performed in parallel but after the initial 2 hour incubation the infected monolayers were washed and 2 ml of MEM containing 100 mg/L gentamicin was added to kill all external bacteria. Plates were incubated for a further 2 h and monolayers disrupted and the cfu/mL determined by serial dilution onto LB agar. Adhesion was calculated as the number of bacteria associated with the eukaryotic cells minus the number that had invaded. Each assay was repeated a minimum of three times, with each repeat including four technical replicates per bacterial strain. The results were analysed using Student’s t-test and *P* values of ≤0.05 were taken as significant.

### Motility Assays

Swimming and swarming motility was measured as previously described [27].

### Measurement of Salmonella Pathogenicity Island (SPI) Gene Expression in Single Cells Using GFP Reporters

Strains containing a chromosomal GFP reporter fused to the promoters of the *ssaG* or *prgH* genes were grown overnight in LB broth at 37°C with shaking. A 4% inoculum was added to 10 ml of either LB, MEM or minimal media and incubated at 37°C with shaking until mid-log phase (OD600 of 0.6). Cells were harvested from 500 µl of culture by centrifugation and re-suspended in 1 ml PBS. 100 µl of each cell suspension was added to a 96 well plate and bacteria were analysed by flow cytometry using an Accuri C6 cytometer. 10 000 data points were collected for each sample.

## Results

### The Transcriptome of *Salmonella* is Medium Dependent

To determine the impact of medium choice, a microarray was carried out to compare the transcriptome of *S*. Typhimurium after growth in LB broth and MOPS minimal medium. A total of 621 genes were differentially expressed between LB and MOPS minimal medium (B >0). Compared to growth in LB broth, 287 genes were increased in expression after growth in MOPS minimal medium (Table S2) whereas 334 were decreased in expression (Table S3). Quantitative real time RT-PCR confirmed gene expression changes for 11 genes whose expression was changed in the microarray (figure S1).

Compared with growth in LB broth, after growth in MOPS minimal medium, the expression of nine genes found in SPI-2 including those encoding apparatus proteins, secreted effectors and a chaperone was increased (Table 1) while only one gene found in SPI-1, *invJ*, had altered expression (2.4 fold increase). In addition, one gene found in SPI-3, *cigR*, showed increased expression by 3.2 fold after growth in MOPS minimal medium and one gene from SPI-6, *safC* had expression increased by 8.06 fold. No SPI genes had reduced expression after growth in minimal media although two virulence related genes, *sseJ* and *ychP*, had decreased expression by 0.3 and 0.7 fold, respectively.

**Table 1:** Genes found in *Salmonella* pathogenicity islands with altered expression after growth of SL1344 in minimal media compared to growth in LB media.

SPI	Gene name	Description	Fold Change	B value
SPI-1	*invJ*	Surface presentation of antigens protein	2.38	3.20
SPI-2	*sseB*	Secretion system effector protein	6.07	8.90
SPI-2	*ssaH*	Secretion system apparatus protein	5.68	11.06
SPI-2	*sseA*	Chaperone protein*	5.22	11.60
SPI-2	*ssaJ*	Secretion system apparatus protein	4.57	7.49
SPI-2	*sscA*	Type III secretion system chaperone protein	3.96	16.90
SPI-2	*ssaR*	Secretion system apparatus protein	3.77	0.74
SPI-2	*sseC*	Secretion system effector protein	3.12	6.92
SPI-2	*sseE*	Secretion system effector protein	2.22	1.14
SPI-3	*cigR*	Exported protein	3.20	10.72
SPI-6	*safC*	Outer membrane fimbrial usher protein	8.06	21.43

Data with a B value (log odds value) >0 and adjusted P value <0.05 were taken as significant.

Compared with LB, growth in MOPS minimal medium also caused numerous gene expression changes in genes involved biosynthesis and transport of amino acids; there was increased expression of 42 genes involved in amino acid synthesis (Table 2). For example, after growth in minimal media there was increased expression of eight genes in the histidine biosynthesis pathway, four genes involved in tryptophan biosynthesis, three genes involved in isoleucine biosynthesis, five genes involved in arginine biosynthesis, three genes involved in leucine biosynthesis, five genes involved in lysine biosynthesis, two genes involved in asparagine biosynthesis, five genes involved in methionine biosynthesis, one gene involved in serine biosynthesis and six genes involved in cysteine biosynthesis (Table 2). In addition, growth in minimal media caused increased expression of 23 ABC transporter genes many of which are involved in amino acid transport (Table 3).

**Table 2:** Genes involved in amino acid biosynthesis that had increased expression after growth of SL1344 in minimal media compared to LB.

	Gene name	Product	Fold change
Histidine biosynthesis	*hisA*	phosphoribosylformimino-5-aminoimidazole carboxamide ribotide isomerase	7.20
	*hisB*	histidinol phosphatase	11.48
	*hisC*	histidinol-phosphate aminotransferase (imidazole	16.41
	*hisF*	cyclase HisF	8.64
	*hisG*	ATP phosphoribosyltransferase	22.72
	*hisH*	amidotransferase	12.87
	*hisI*	phosphoribosyl-AMP cyclohydrolase/phosphoribosyl-ATP pyrophosphohydrolase	12.39
	*hisJ*	histidine-binding periplasmic protein	4.00
Tryptophan biosynthesis	*trpA*	tryptophan synthase alpha chain	9.41
	*trpB*	tryptophan synthase beta chain	11.86
	*trpC*	indole-3-glycerol phosphate synthase	7.15
	*trpD*	anthranilate synthase component II; anthranilate phosphoribosyltransferase	5.54
Isoleucine Biosynthesis	*asd*	aspartate-semialdehyde dehydrogenase	5.10
	*ilvN*	acetohydroxy acid synthase I, small subunit	2.35
	*thrB*	homoserine kinase	5.15
Arginine biosynthesis	*argA*	N-acetylglutamate synthase	3.57
	*argC*	N-acetyl-gamma-glutamyl-phosphate reductase	5.35
	*argD*	acetylornithine aminotransferase	7.62
	*argH*	argininosuccinate lyase	10.42
	*argT*	lysine-arginine-ornithine-binding periplasmic protein precursor	19.27
Leucinebiosynthesis	*leuA*	2-isopropylmalate synthase	4.12
	*leuB*	3-isopropylmalate dehydrogenase	9.22
	*leuC*	3-isopropylmalate dehydratase	7.77
Lysine biosynthesis	*lysA*	diaminopimelate decarboxylase	6.96
	*lysC*	lysine-sensitive aspartokinase III	8.80
	*argD*	acetylornithine aminotransferase	7.62
	*dapB*	dihydrodipicolinate reductase	5.85
	*asd*	aspartate-semialdehyde dehydrogenase	5.10
Asparagine biosynthesis	*asnA*	asparagine synthetase A	18.51
	*asnB*	asparagine synthetase B	5.05
Methionine biosynthesis	*metA*	homoserine O-succinyltransferase	2.56
	*metC*	beta-cystathionase	1.66
	*metF*	5,10 methylenetetrahydrofolate reductase	2.94
	*Meth*	12-dependent homocysteine-N5- methyltetrahydrofolate transmethylase	2.25
	*asd*	aspartate-semialdehyde dehydrogenase	5.10
Serine biosynthesis	*serC*	phosphoserine aminotransferase	3.50
Cysteine biosynthesis	*cysA*	sulphate transport ATP-binding protein CysA	14.83
	*cysH*	3'-phosphoadenosine 5'-phosphosulfate sulfotransferase	5.33
	*cysI*	sulfite reductase (NADPH) hemoprotein alpha subunit	5.26
	*cysJ*	sulfite reductase (NADPH) hemoprotein alpha subunit	11.28
	*cysK*	cysteine synthase A	7.82
	*cysM*	cysteine synthase B	3.26

**Table 3:** ABC Transporter genes with increased expression after growth of SL1344 in minimal media.

	Gene	Product	Fold change
Oligopeptide transport	*oppA*	periplasmic oligopeptide-binding protein precursor	3.90
	*oppB*	oligopeptide transport system permease protein OppB	3.25
	*oppC*	oligopeptide transport system permease protein OppC (pseudogene)	3.23
	*oppF*	oligopeptide transport ATP-binding protein OppF	2.56
Dipeptide transport	*dppA*	periplasmic dipeptide transport protein precursor	17.45
	*dppB*	dipeptide transport system permease protein DppB	19.62
	*dppC*	dipeptide transport system permease protein DppC	15.64
	*dppD*	dipeptide transport ATP-binding protein DppD	12.86
Histidine transport	*hisJ*	histidine-binding periplasmic protein	4.00
	*hisM*	histidine transport system permease	4.40
	*hisP*	histidine transport ATP-binding protein	5.87
	*hisQ*	histidine transport system permease protein	4.78
Leucine transport	*livH*	high-affinity branched-chain amino acid transport system permease protein	1.73
	*livJ*	amino acid-binding protein	5.68
	*livK*	leucine-specific binding protein	3.57
	*livM*	high-affinity branched-chain amino acid transport system permease protein	1.56
Arginine Transport	*artM*	arginine transport system permease protein ArtM	1.88
	*artP*	arginine transport ATP-binding protein ArtP	2.46
	*artQ*	arginine transport system permease protein ArtQ	2.42
Putrescine transport	*potG*	putrescine transport ATP-binding protein PotG	4.15
	*potH*	putrescine transport system permease protein PotH	2.34
Sulphate transport	*cysA*	sulphate transport ATP-binding protein CysA	14.83
	*cysU*	sulphate transport system permease protein CysT	9.77

**Figure 1:**
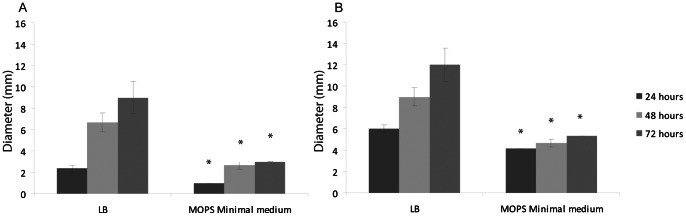
Swimming (A) and Swarming (B) Motility of SL1344 was reduced on MOPS minimal medium. Data are displayed as a mean of at least three separate experiments +/− standard deviation.

After growth in MOPS minimal media seven flagellar genes had decreased expression. This correlated with a decrease in both swimming and swarming motility on MOPS minimal semi-solid agar compared to LB semi-solid agar (Figure 1).

### Overnight Growth in MOPS Minimal Medium Increases Adherence of *Salmonella* to INT-407 Cells *in vitro*

**Figure 2:**
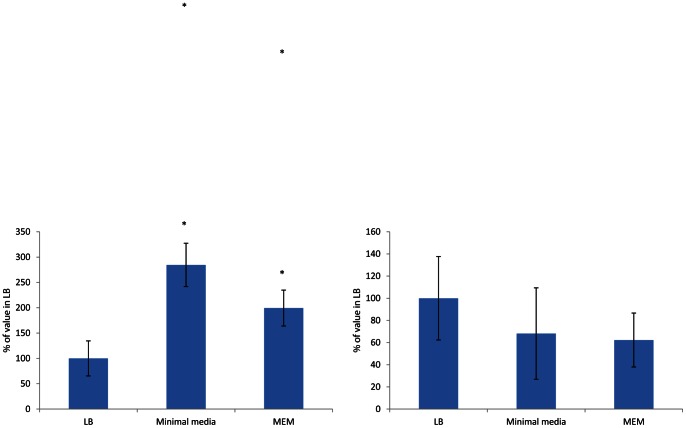
Adhesion (A) and Invasion (B) of SL1344 into INT-407 after growth in LB broth, MEM or MOPS minimal medium. Data are displayed as mean of at least three separate experiments performed in triplicate + standard deviation. For each strain values after growth in MEM or MOPS minimal medium were compared to the value for LB. Values returning a p value of ≤0.05 from a Student's T-test are denoted by *.

Adhesion of *S.* Typhimurium SL1344 to INT-407 cells was significantly increased after overnight growth in MOPs minimal media compared to overnight growth in LB (*P* = 0.04) (Figure 2). Invasion of INT-407 cells by SL1344 was not significantly altered by overnight growth in MOPs minimal media.

### Growth in MEM Increases Adhesion and Expression of Genes in SPI-2

Growth in MOPS minimal medium altered ability to adhere to eukaryotic cells compared with growth in LB and impacted the expression of genes related to virulence. In bacteriology, many assays that measure attributes of virulence, including adhesion and invasion, use tissue culture media such as Minimal Essential Medium (MEM). Therefore, the effect of growth in MEM was assessed to inform experimental design of these assays and to deduce the biological effect of this medium choice.

Adhesion of *S.* Typhimurium SL1344 to INT-407 cells was also significantly increased after overnight growth in MEM compared to overnight growth in LB (*P* = 0.05) although the effect was less pronounced than that seen after growth in MOPS minimal medium (Figure 2). Invasion of INT-407 cells by SL1344 was not significantly altered by overnight growth in MEM.

After growth in MEM the expression of selected genes, whose expression was altered after growth in MOPS minimal medium, was measured to see whether expression changes were similar in the two medium types and if this could explain the differences in infection phenotype. As many SPI-2 genes had altered expression after growth in MOPs minimal medium, three genes from this group were selected including a chaperone (*sseA*), an effector protein (*sseB*) and a component of the T3SS apparatus (*ssaH*). The microarray and RT-PCR both showed that each of these genes had increased expression after growth in MOPS minimal medium compared to LB broth. After growth in MEM the expression of *sseA*, *sseB* and *sseH* was also increased compared to LB; expression of *sseA* and *sseB* was increased to a similar extent in both MOPS minimal medium and MEM but *sseH* was expressed at a higher level in MEM than in MOPS minimal medium or LB broth (Table 4).

**Table 4:** Expression of SPI-2 genes is increased by growth in MEM or Minimal media.

Gene	Media	Normalised Fold Expression Relative to LB	
		Microarray data	Real-time RT-PCR
*sseA* (SPI-2 chaperone protein)	MEM		5.86
	Min	5.22	4.94
*sseB* (SPI-2 secretion system effector protein)	MEM		3.09
	Min	6.07	2.00
*ssaH* (SPI-2 secretion system apparatus protein)	MEM		2.18
	Min	5.68	11.76

### Medium Type Alters the Proportion of the Population Expressing SPI-1 or SPI-2

**Figure 3:**
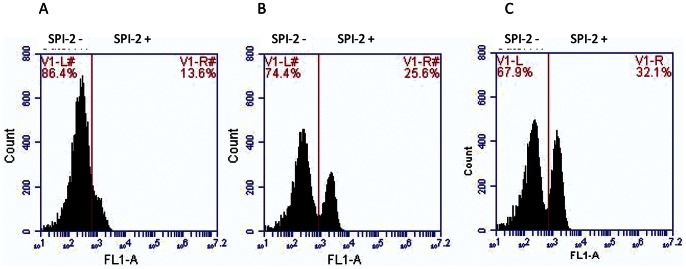
Growth in MEM or MOPS minimal medium increased the proportion of the population expressing SPI-2. Representative flow cytometry spectra showing percentage of cells expressing GFP after growth in LB (A), MOPS minimal medium (B) and MEM (C).

Genes from SPI-1 and SPI-2 are expressed by only a portion of the total population [10], [11], [12] so techniques such as microarrays and RT-PCR, which measure gene expression across a whole population, give only partial information about gene expression in the bacterial population. Strains containing promoters of either *prgH* (SPI-1) or *ssaG* (SPI-2) fused to *gfp* were used to compare the percentage of cells in the population expressing SPI-1 or SPI-2 in different media using flow cytometry. After growth to mid logarithmic phase in LB broth 17.52% of the SL1344 population expressed SPI-1-GFP and 14.25% expressed SPI-2-GFP. In comparison, growth in MEM or MOPS minimal medium did not significantly alter the percentage of cells expressing SPI-1. However, the percentage of the bacterial population expressing SPI-2 was significantly increased after growth in either MEM or MOPS minimal medium (33.53% and 26.8%, respectively) (Figure 3).

## Discussion

The transcriptomic differences after growth of *S.* Typhimurium to mid-logarithmic phase in different media were profound. For example, there was increased expression of many genes involved in amino acid biosynthesis and transport after growth in MOPS minimal medium. This is not un-expected as there are fewer amino acids in the MOPS minimal medium than in the rich medium so during growth in MOPS minimal medium the relevant metabolic pathways are up-regulated to synthesise and transport the necessary precursor molecules. This finding is similar to that of Tao and colleagues who found increased expression of amino acid biosynthetic pathways in *Escherichia coli* after growth in M63 minimal medium [28]. Other patterns detected in the transcriptomic data resulted in measurable phenotypic differences. For example, after growth in MOPS minimal medium there was decreased expression of several genes involved in motility and this corresponded with decreased swimming and swarming motility on MOPS minimal medium compared to LB medium. Additionally, growth in MOPS minimal medium or MEM increased expression of SPI-2 and altered the adhesive ability of Salmonella to INT-407 cells growing in tissue culture. The increase in adhesion of SL1344 after growth in minimal media may be due to altered expression of fimbrial adhesins as the *safC* gene encoding the small outer membrane fimbrial usher protein had increased expression by 8.06 fold after growth in minimal media (Table 1). However, further work would be required to confirm this link. The lack of change in expression of genes in SPI-1 correlates with the level of invasion being unaltered.

Expression of SPI-1 and SPI-2 are controlled in response to environmental cues that signal when these systems are required. *In vivo* SPI-2 expression is increased when *Salmonella* are intracellular to allow intracellular survival and replication [29]. Interestingly, the same SPI-2 genes with increased expression in MOPS minimal medium (Table 1) were also increased in expression after replication of *Salmonella* Typhimurium SL1344 in murine macrophages (between 5.96 and 10.68 fold) [8]. Concentrations of Ca^2+^ or and Mg^2+^ below 8 µM were shown to induce SPI-2 expression [16]. The Ca^2+^ concentration inside macrophage lysosomes has been estimated to be around 400 µM (at least five times lower than the extracellular concentration of 2 mM) and MOPS minimal medium contains much less Ca^2+^ than this (∼0.5 µM) [20]. Therefore, we hypothesise that increased SPI-2 expression after growth in minimal media could be due to the very low Ca^2+^ concentration [18], [30]. However, the concentration of Mg^2+^ in MOPS minimal medium is 500 µM, 2.5 times higher than in LB, so Mg^2+^ is unlikely to be a contributory factor [20].

The microarray study and confirmatory RT-PCRs showed increased SPI-2 transcript levels. These techniques, and those using reporter constructs in studies cited above, measure the transcript level across the whole population. However, expression of SPI genes is bimodal [10], . Here, for the first time, we have studied the effect of medium choice on SPI-2 expression at the individual cell level. SL1344 containing GFP reporter constructs to measure SPI-1 and SPI-2 expression were grown in the different types of medium and then analysed by flow cytometry. This showed that the reported increase in population transcript level related to an increased proportion of the population expressing the SPI-2 genes. The finding that the SPI-2 induction by environmental conditions does not affect the whole population in the same way is important information for experimental design of future studies of *Salmonella* phenotype and virulence gene expression. Furthermore, not only is the medium choice important but when studying expression of pathogenicity island genes, techniques such as flow cytometry which allow quantification of expression on the single cell level as well as studies at the whole population level, should be used.

In summary, our study shows that the impact of medium choice on the transcriptome of the model pathogen Salmonella is profound and therefore, the choice of laboratory medium is an important consideration for all microbiological studies.

## Supporting Information

Figure S1 (TIF)

Table S1 Primers used in this study. (DOC)

Table S2 Groups of S. Typhimurium genes with increased expression after growth in MOPS minimal medium. (DOC)

Table S3 Groups of S. Typhimurium genes with decreased expression after growth in MOPS minimal medium. (DOC)
