# Supplementary figures and images for: Choice of Bacterial Growth Medium Alters the Transcriptome and Phenotype of Salmonella enterica Serovar Typhimurium
Source: PLoS One. 2013 May 21;8(5):e63912. doi: 10.1371/journal.pone.0063912 (PMC3660369; doi:10.1371/journal.pone.0063912)

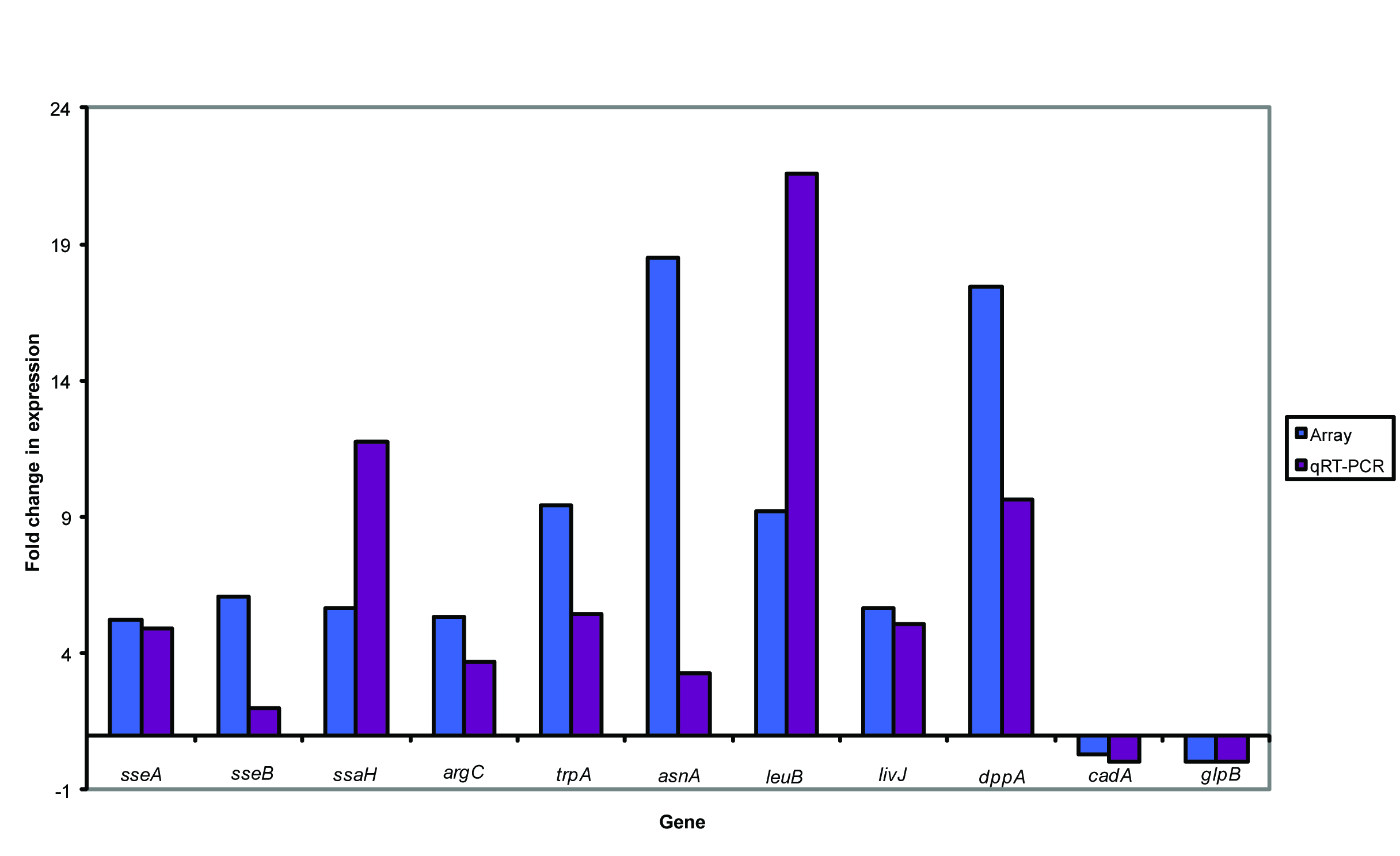

Supplement: Figure S1 — (TIF) [file pone.0063912.s001.tif]
