# Supplementary material for: Choice of Bacterial Growth Medium Alters the Transcriptome and Phenotype of Salmonella enterica Serovar Typhimurium
Source: PLoS One. 2013 May 21;8(5):e63912. doi: 10.1371/journal.pone.0063912 (PMC3660369; doi:10.1371/journal.pone.0063912)
Supplement: Table S1 — (DOC) [file pone.0063912.s002.doc]

**Supplementary table 1. Primers used in this study**

| **Gene** | **Primer Sequence** | |
| --- | --- | --- |
|  | **Forward** | **Reverse** |
| *sseA* | GGGCTAAGGTGAGTCAACA | TGAAGAATACTCTCTGTCTCTCTG |
| *sseB* | CATCTTATGGGGAAGTCAAAACC | GATAAGTCATCCTGGCTCCC |
| *ssah* | TGATTTCCCAGGTACATGCGATG | GCCAACAATAATGCCAGACATACC |
| *trpA* | CAGCCATTGTCAAGATTATCG | CTGAGACAAAGGACCTGAG |
| *argC* | CAGTTTCTGTGAAGTGAGTTT | CAGATGGACGGCGATTTC |
| *leuB* | AAGCCTTTGATACCGAAG | CTTTATCAATAGAGGTGACTTT |
| *asnA* | GTCTATGTGGATCAGTGG | ATATAGCCTCTACTGTGC |
| *dppA* | ATCAAAGCCGTTTATCAG | TTAATATCGTCGTTGTAGC |
| *artP* | GTCTTTCAGCAATATAATCTTTGG | TCTTTTGTCAGACCCAGTA |
| *livJ* | CAACGGCGGCAAAGTATA | CGTACTGCTGCTTATCGT |
| *cadA* | TAAATCGCTGACCCATCT | GAATACCGCCAAGAATACC |
| *glpB* | GTATCAACAACATTTCTT | GCATACAGATTTTCTACC |
