# Supplementary material for: Choice of Bacterial Growth Medium Alters the Transcriptome and Phenotype of Salmonella enterica Serovar Typhimurium
Source: PLoS One. 2013 May 21;8(5):e63912. doi: 10.1371/journal.pone.0063912 (PMC3660369; doi:10.1371/journal.pone.0063912)
Supplement: Table S2 — (DOC) [file pone.0063912.s003.doc]

**Supplementary Table 2. Groups of *S*. Typhimurium genes with increased expression after growth in MOPS minimal medium**

|  | **Gene name** | **Description** | **Fold change** |
| --- | --- | --- | --- |
| Invasion related genes | | *pagC* | | --- | | *uvrY* | | *invJ* | | *sseB* | | *ssaH* | | *sseA* | | *ssaJ* | | *sscA* | | *ssaR* | | *sseC* | | *sseE* | | *cigR* | | *safC* | | | outer membrane invasion protein | | --- | | invasion response-regulator | | surface presentation of antigens protein | | pathogenicity island effector effector protein | | pathogenicity island protein | | Chaperone protein | | pathogenicity island lipoprotein | | Type III secretion system chaperone protein | | type III secretion protein | | pathogenicity island effector protein | | pathogenicity island effector protein | | exported protein | | outer-membrane fimbrial usher protein | | | 21.23 | | --- | | 2.37 | | 2.38 | | 6.07 | | 5.68 | | 5.22 | | 4.57 | | 3.96 | | 3.77 | | 3.12 | | 2.22 | | 3.20 | | 8.06 | |
| **Metabolism Genes** | | *glnK* | | --- | | *cueO* | | *hisG* | | *argT* | | *asnA* | | *hisC* | | *hisH* | | *hisI* | | *trpB* | | *hisB* | | *cysJ* | | *glnH* | | *argH* | | *trpA* | | *leuB* | | *lysC* | | *hisF* | | *gdhA* | | *cysK* | | *leuC* | | *argD* | | *copA* | | *hisA* | | *glnA* | | *trpC* | | *ilvI* | | *lysA* | | *manZ* | | *yjeM* | | *dapB* | | *ccmF* | | *aroG* | | *yfbE* | | *livJ* | | *trpD* | | *argC* | | *cysH* | | *manY* | | *cysI* | | *thrB* | | *asd* | | *asnB* | | *ccmG* | | *yfcF* | | *gltA* | | *leuA* | | *sucD* | | *glgA* | | *hisJ* | | *sdhD* | | *oppA* | | *purT* | | *xerC* | | *livK* | | *argA* | | *narI* | | *serC* | | *yneB* | | *malP* | | *malQ* | | *carB* | | *ppc* | | *folA* | | *cysM* | | *sucB* | | *pfkB* | | *carA* | | *gst* | | *metF* | | *glgP* | | *yjcD* | | *pntB* | | *nadA* | | *yicE* | | *metA* | | *aegA* | | *napA* | | *purN* | | *glgB* | | *ilvN* | | *yfbT* | | *tag* | | *pyrC* | | *metH* | | *mtlA* | | *nirD* | | *gcvT* | | *ndk* | | *rfbI* | | *pykA* | | *cbiH* | | *argI* | | *otsA* | | *ygcA* | | *ribB* | | *aldB* | | *grxA* | | *yedO* | | *purK* | | *grxB* | | *livH* | | *cbiC* | | *yfdZ* | | *nrfC* | | *mtlD* | | *metC* | | *yieH* | | *cbiK* | | *slt* | | *cbiJ* | | *cobT* | | *relA* | | *cbiF* | | *adi* | | *solA* | | *rtcA* | | *manX* | | *srlE* | | *eutS* | | | nitrogen regulatory protein P-II | | --- | | possible multicopper oxidase precursor | | ATP phosphoribosyltransferase | | lysine-arginine-ornithine-binding periplasmic protein precursor | | asparagine synthetase A | | histidinol-phosphate aminotransferase (imidazole) | | amidotransferase | | phosphoribosyl-AMP cyclohydrolase / phosphoribosyl-ATP pyrophosphohydrolase | | tryptophan synthase beta chain | | imidazoleglycerol-phosphate dehydratase; histidinol phosphatase | | sulfite reductase (NADPH) flavoprotein beta subunit | | glutamine-binding periplasmic protein precursor | | argininosuccinate lyase | | tryptophan synthase alpha chain | | 3-isopropylmalate dehydrogenase | | lysine-sensitive aspartokinase III | | cyclase HisF | | NADP-specific glutamate dehydrogenase | | cysteine synthase A | | 3-isopropylmalate dehydratase | | acetylornithine aminotransferase | | copper-transporting ATPase | | phosphoribosylformimino-5-aminoimidazole carboxamide ribotide isomerase | | glutamine synthetase | | indole-3-glycerol phosphate synthase | | acetolactate synthase isozyme III large subunit | | diaminopimelate decarboxylase | | phosphotransferase enzyme II, D component | | putative amino acid permease | | dihydrodipicolinate reductase | | cytochrome c-type biogenesis protein F1 | | phospho-2-dehydro-3-deoxyheptonate aldolase | | putative lipopolysaccharide biosynthesis protein | | amino acid-binding protein (pseudogene) | | anthranilate synthase component II; anthranilate phosphoribosyltransferase | | N-acetyl-gamma-glutamyl-phosphate reductase | | 3'-phosphoadenosine 5'-phosphosulfate sulfotransferase | | phosphotransferase enzyme II, C component | | sulfite reductase (NADPH) hemoprotein alpha subunit | | homoserine kinase | | aspartate-semialdehyde dehydrogenase | | asparagine synthetase B | | thiol:disulfide interchange protein | | putative glutathione-S transferase | | citrate synthase | | 2-isopropylmalate synthase | | succinyl-CoA synthetase alpha chain | | glycogen synthase | | histidine-binding periplasmic protein | | succinate dehydrogenase hydrophobic membrane anchor protein | | periplasmic oligopeptide-binding protein precursor | | phosphoribosylglycinamide formyltransferase 2 | | integrase/recombinase | | leucine-specific binding protein | | N-acetylglutamate synthase | | respiratory nitrate reductase 1 gamma chain | | phosphoserine aminotransferase | | putative aldolase | | maltodextrin phosphorylase | | 4-alpha-glucanotransferase | | carbamoyl-phosphate synthase large chain | | phosphoenolpyruvate carboxylase | | dihydrofolate reductase type I | | cysteine synthase B | | dihydrolipoamide succinyltransferase component | | 6-phosphofructokinase isozyme | | carbamoyl-phosphate synthase small chain | | glutathione S-transferase | | 5,10 methylenetetrahydrofolate reductase | | glycogen phosphorylase | | putative xanthine/uracil permeases family protein | | pyridine nucleotide transhydrogenase subunit- beta | | quinolinate synthetase A protein | | putative purine permease | | homoserine O-succinyltransferase | | putative oxidoreductase | | probable nitrate reductase | | phosphoribosylglycinamidine myltransferase | | 1,4-alpha-glucan branching enzyme | | acetohydroxy acid synthase I, small subunit | | putative phosphatase | | 3-methyladenine DNA glycosylase I, constitutive | | dihydroorotase | | B12-dependent homocysteine-N5- methyltetrahydrofolate transmethylase | | mannitol-specific enzyme II of phosphotransferase system | | nitrite reductase (NAD(P)H) small subunit | | aminomethyltransferase | | nucleoside diphosphate kinase (ndk) | | bactoprenol-linked glucose translocase | | pyruvate kinase A | | precorrin-3 C17-methyltransferase | | ornithine carbamoyltransferase | | trehalose-6-phosphate synthase | | putative RNA methyltransferase | | 3,4-dihydroxy-2-butanone 4-phosphate synthase | | aldehyde dehydrogenase B | | glutaredoxin 1 | | putative deaminase | | phosphoribosylaminoimidazole carboxylase ATPase subunit | | glutaredoxin 2 | | high-affinity branched-chain amino acid transport system permease protein | | precorrin-8X methylmutase (pseudogene) | | putative aminotransferase | | cytochrome c-type biogenesis protein | | mannitol-1-phosphate dehydrogenase | | beta-cystathionase | | putative hydrolase | | putative cobalamin biosynthethesis protein (pseudogene) | | lytic murein transglycosylase | | precorrin-6x reductase (pseudogene) | | nicotinate-nucleotide--dimethylbenzimidazole phosphoribosyl transferase | | GTP pyrophosphokinase | | precorrin-4 C11-methyltransferase | | arginine decarboxylase | | putative sarcosine oxidase | | RNA 3'-terminal phosphate cyclase (with b3419) | | PTS system, mannose-specific IIAB component | | PTS system, glucitol/sorbitol-specific IIBC component | | putative ethanolamine utilization protein EutS | | | 38.32 | | --- | | 25.17 | | 22.72 | | 19.27 | | 18.51 | | 16.41 | | 12.87 | | 12.39 | | 11.86 | | 11.48 | | 11.28 | | 10.55 | | 10.42 | | 9.41 | | 9.22 | | 8.80 | | 8.64 | | 7.84 | | 7.82 | | 7.77 | | 7.62 | | 7.41 | | 7.20 | | 7.18 | | 7.15 | | 6.99 | | 6.96 | | 6.81 | | 5.94 | | 5.85 | | 5.82 | | 5.81 | | 5.80 | | 5.68 | | 5.54 | | 5.35 | | 5.33 | | 5.33 | | 5.26 | | 5.15 | | 5.10 | | 5.05 | | 4.35 | | 4.26 | | 4.24 | | 4.12 | | 4.10 | | 4.01 | | 4.00 | | 3.91 | | 3.90 | | 3.81 | | 3.78 | | 3.57 | | 3.57 | | 3.54 | | 3.50 | | 3.45 | | 3.42 | | 3.41 | | 3.35 | | 3.35 | | 3.32 | | 3.26 | | 3.22 | | 3.12 | | 3.02 | | 2.94 | | 2.94 | | 2.78 | | 2.70 | | 2.65 | | 2.59 | | 2.57 | | 2.56 | | 2.56 | | 2.44 | | 2.37 | | 2.36 | | 2.35 | | 2.34 | | 2.29 | | 2.28 | | 2.25 | | 2.23 | | 2.18 | | 2.07 | | 2.07 | | 2.04 | | 2.04 | | 1.95 | | 1.93 | | 1.89 | | 1.87 | | 1.86 | | 1.85 | | 1.82 | | 1.79 | | 1.78 | | 1.74 | | 1.73 | | 1.73 | | 1.70 | | 1.68 | | 1.67 | | 1.66 | | 1.65 | | 1.64 | | 1.63 | | 1.63 | | 1.55 | | 1.54 | | 1.52 | | 1.49 | | 1.49 | | 1.35 | | 3.59 | | 2.55 | | 1.55 | |
| **Outer membrane proteins** | | *ompW* | | --- | | *ompF* | | | outer membrane protein | | --- | | outer membrane protein F precursor | | | 5.37 | | --- | | 31.79 | |
| **Transport genes** | | *gltI* | | --- | | *amtB* | | *gltK* | | *dppB* | | *dppA* | | *dppC* | | *cysA* | | *dppD* | | *aroP* | | *cysU* | | *sfbC* | | *sfbB* | | *hisP* | | *hisQ* | | *hisM* | | *potG* | | *yecC* | | *yhjE* | | *oppB* | | *oppC* | | *yieG* | | *modF* | | *oppF* | | *ydjN* | | *artP* | | *artQ* | | *potH* | | *fepE* | | *artM* | | *abc* | | *putP* | | *livM* | | *ycaD* | | *phnU* | | *malK* | | *cyaY* | | | ABC transporter periplasmic binding protein | | --- | | ammonium transporter | | glutamate/aspartate transport system permease protein GltK | | dipeptide transport system permease protein DppB | | periplasmic dipeptide transport protein precursor | | dipeptide transport system permease protein DppC | | sulphate transport ATP-binding protein CysA | | dipeptide transport ATP-binding protein DppD | | aromatic amino acid transport protein AroP | | sulphate transport system permease protein CysT | | ABC transporter integral membrane protein | | ABC transporter ATP-binding protein | | histidine transport ATP-binding protein | | histidine transport system permease protein | | histidine transport system permease | | putrescine transport ATP-binding protein PotG | | putative ABC-transport ATP-binding protein | | hypothetical metabolite transport protein | | oligopeptide transport system permease protein OppB | | oligopeptide transport system permease protein OppC (pseudogene) | | membrane transport protein | | molybdenum transport ATP-binding protein | | oligopeptide transport ATP-binding protein OppF | | putative sodium:dicarboxylate symporter | | arginine transport ATP-binding protein ArtP | | arginine transport system permease protein ArtQ | | putrescine transport system permease protein PotH | | ferric enterobactin transport protein FepE (pseudogene) | | arginine transport system permease protein ArtM | | ABC transporter ATP-binding protein | | sodium/proline symporter (proline permease) | | high-affinity branched-chain amino acid transport system permease protein | | probable transport protein | | probable membrane component of 2- aminoethylphosphonate transporter | | maltose/maltodextrin transport ATP-binding protein | | CyaY protein - transport | | | 39.20 | | --- | | 32.27 | | 25.14 | | 19.62 | | 17.45 | | 15.64 | | 14.83 | | 12.86 | | 12.15 | | 9.77 | | 9.16 | | 8.60 | | 5.87 | | 4.78 | | 4.40 | | 4.15 | | 3.99 | | 3.80 | | 3.25 | | 3.23 | | 2.68 | | 2.63 | | 2.56 | | 2.48 | | 2.46 | | 2.42 | | 2.34 | | 2.23 | | 1.88 | | 1.67 | | 1.59 | | 1.56 | | 1.55 | | 1.54 | | 1.36 | | 2.75 | |
| **Regulatory genes** | | *cpxR* | | --- | | *glnG* | | *phoH* | | *creB* | | | two-component response regulatory protein | | --- | | Two-component system, response regulator | | PhoH protein (phosphate starvation-inducible protein) | | putative two-component response regulator | | | 2.50 | | --- | | 2.45 | | 1.84 | | 1.72 | |
| **Transcriptional regulatory genes** | | *lrhA* | | --- | | *fimZ* | | *xapR* | | *ptsJ* | | *ybeF* | | *tdcA* | | | NADH dehydrogenase operon transcription regulator | | --- | | probable transcriptional regulator (FimXZ protein) | | xanthosine operon transcriptional regulator | | putative transcriptional regulator | | lysR-family transcriptional regulator | | TDC operon transcriptional activator | | | 3.10 | | --- | | 2.95 | | 2.07 | | 1.95 | | 1.83 | | 1.64 | |
| **Lipoprotein associated genes** | | *sfbA* | | --- | | *lpp* | | *nlpC* | | *lppB* | | *pal* | | *envF* | | | lipoprotein | | --- | | major outer membrane lipoprotein | | putative lipoprotein | | major outer membrane lipoprotein | | peptidoglycan-associated lipoprotein precursor | | putative envelope lipoprotein | | | 18.85 | | --- | | 3.33 | | 3.01 | | 2.67 | | 2.48 | | 2.00 | |
| Plasmid genes | | *traS* | | --- | | *traY* | | *trbD* | | *trbC* | | *traT* | | *traT* | | *traA* | | | conjugative transfer: surface exclusion | | --- | | conjugative transfer: oriT nicking | | conjugative transfer: | | conjugative transfer: assembly | | conjugative transfer: surface exclusion | | TraT protein | | conjugative transfer: fimbrial subunit | | | 3.65 | | --- | | 2.67 | | 1.61 | | 1.43 | | 7.19 | | 5.34 | | 9.41 | |
| **Phage genes** | | *sodC* | | --- | | STM2585 | | STM2590 | | | Gifsy-2 prophage | | --- | | Gifsy-1 prophage | | Gifsy-1 prophage | | | 5.26 | | --- | | 2.82 | | 2.35 | |
| **Genes with unknown, miscellaneous or putative function** | | STM1410 | | --- | | *yjaH* | | STM2780 | | *sbp* | | STM2359 | | *ydiH* | | *pmrF* | | STM2178 | | *yebE* | | *ugtL* | | *yccA* | | STM2300 | | STM0353 | | *cvpA* | | *cvpA* | | STM0354 | | *yciW* | | *malM* | | *ycfS* | | *scsA* | | *fliY* | | *ydiQ* | | *yrfF* | | *yigA* | | *yaiB* | | STM1733 | | *lamB* | | STM4002 | | STM1130 | | STM1132 | | STM1676 | | STM3944 | | *htpX* | | *napG* | | STM2139 | | STM2336 | | *ratA* | | *yafK* | | STM1857 | | STM1669 | | STM1366 | | *yfbU* | | STM0292 | | STM1133 | | STM2745 | | *yiiL* | | STM4509 | | STM1541 | | STM2752 | | *ynfD* | | *yigZ* | | *ydaA* | | STM2749 | | *ybdQ* | | STM3142 | | *yeiB* | | *yciI* | | STM1600 | | STM2955 | | STM1864 | | STM1273 | | *yeaH* | | STM1368 | | STM3772 | | STM4351 | | STM1131 | | STM2942 | | STM0290 | | *pduG* | | STM3771 | | *yciC* | | *ymgE* | | *yecN* | | *ylbA* | | STM4272 | | *yjbA* | | STM0947 | | *yohM* | | *yfeH* | | STM1618 | | STM3768 | | STM4447 | | *yfcH* | | STM1324 | | *ygiC* | | *ydaL* | | mreD | | *ygcH* | | *Fic* | | *yicN* | | | putative pathogenicity island protein | | --- | | conserved hypothetical protein | | conserved hypothetical protein | | periplasmic sulphate binding protein | | putative amino acid transporter | | conserved hypothetical protein | | putative lipopolysaccharide modification protein | | putative gentisate 1,2-dioxygenase | | conserved hypothetical protein | | putative membrane protein | | putative membrane protein | | conserved hypothetical protein | | putative cation transport ATPase | | colicin V production protein (DedE protein) | | colicin V production protein (DedE protein) | | putative transcriptional regulator | | conserved hypothetical protein | | maltose operon periplasmic protein | | putative exported protein | | membrane protein, suppressor for copper- sensitivity A | | cystine-binding periplasmic protein precursor | | putative electron transfer flavoprotein | | putative membrane protein | | conserved hypothetical protein | | conserved hypothetical protein | | conserved hypothetical protein | | maltoporin precursor | | hypothetical protein | | conserved hypothetical protein | | putative transporter | | putative oxidoreductase | | putative membrane protein | | heat shock protein | | ferredoxin-type protein NapG | | putative membrane protein | | conserved hypothetical protein | | putative outer membrane protein | | putative exported protein | | putative acetyltransferase | | invasin-like protein | | conserved hypothetical protein | | conserved hypothetical protein | | putative RHS-family protein | | putative oxidoreductase | | putative inner membrane protein | | conserved hypothetical protein | | conserved hypothetical protein | | putative regulatory protein | | putative PTS enzyme III glucitol | | conserved hypothetical protein (pseudogene) | | conserved hypothetical protein | | conserved hypothetical protein | | putative cytoplasmic protein | | conserved hypothetical protein | | possible ABC-transport protein, periplasmic- binding component | | putative membrane protein | | conserved hypothetical protein | | conserved hypothetical protein | | hypothetical protein | | putative membrane protein | | putative membrane protein | | conserved hypothetical protein | | putative transporter | | putative PTS system protein | | probable arginine-binding periplasmic protein | | putative secreted protein | | putative transposase | | hypothetical protein | | PduG protein | | putative PTS system protein | | putative membrane protein | | putative membrane protein | | putative membrane protein | | conserved hypothetical protein | | putative membrane protein | | putative membrane protein | | putative integrase protein | | conserved hypothetical protein (pseudogene) | | putative membrane protein | | putative regulatory protein | | putative transferase | | hypothetical protein | | conserved hypothetical protein | | conserved hypothetical protein | | conserved hypothetical protein | | conserved hypothetical protein | | rod shape-determining protein | | putative cytoplasmic protein | | cell filamentation protein Fic | | putative exported protein | | | 12.87 | | --- | | 11.54 | | 9.98 | | 7.72 | | 6.73 | | 5.92 | | 5.87 | | 5.44 | | 5.18 | | 4.81 | | 4.77 | | 4.69 | | 4.48 | | 4.26 | | 4.26 | | 3.72 | | 3.32 | | 3.14 | | 2.93 | | 2.87 | | 2.86 | | 2.82 | | 2.78 | | 2.74 | | 2.71 | | 2.71 | | 2.67 | | 2.64 | | 2.59 | | 2.54 | | 2.44 | | 2.39 | | 2.38 | | 2.38 | | 2.38 | | 2.32 | | 2.28 | | 2.27 | | 2.25 | | 2.24 | | 2.22 | | 2.21 | | 2.18 | | 2.17 | | 2.13 | | 2.13 | | 2.10 | | 2.08 | | 2.07 | | 2.05 | | 2.04 | | 2.01 | | 2.01 | | 2.00 | | 1.98 | | 1.97 | | 1.95 | | 1.95 | | 1.91 | | 1.91 | | 1.89 | | 1.89 | | 1.86 | | 1.84 | | 1.82 | | 1.79 | | 1.79 | | 1.78 | | 1.77 | | 1.71 | | 1.70 | | 1.69 | | 1.68 | | 1.65 | | 1.64 | | 1.64 | | 1.63 | | 1.62 | | 1.62 | | 1.61 | | 1.60 | | 1.55 | | 1.54 | | 1.51 | | 1.51 | | 1.51 | | 1.44 | | 1.42 | | 1.39 | | 1.33 | |

Genes changes with B>0 were taken as significant
