# Supplementary material for: Choice of Bacterial Growth Medium Alters the Transcriptome and Phenotype of Salmonella enterica Serovar Typhimurium
Source: PLoS One. 2013 May 21;8(5):e63912. doi: 10.1371/journal.pone.0063912 (PMC3660369; doi:10.1371/journal.pone.0063912)
Supplement: Table S3 — (DOC) [file pone.0063912.s004.doc]

**Supplementary Table 3. Groups of *S*. Typhimurium genes with decreased expression after growth in MOPS minimal medium**

|  | **Gene name** | **Description** | **Fold Change** |
| --- | --- | --- | --- |
| Invasion related genes | | *sseJ* | | --- | | *ychP* | | | Salmonella translocated effector: regulated by SPI-2 | | --- | | invasin | | | 0.30 | | --- | | 0.71 | |
| **Fimbrial genes** | bcfE | fimbrial subunit | 0.63 |
| Chemotaxis and motility genes | | *flgA* | | --- | | *fliF* | | *fliH* | | *fliJ* | | *fliO* | | *fliP* | | STM3152 | | *yaiU* | | | flagellar basal body P-ring protein FlgA precursor | | --- | | flagellar basal-body M-ring protein | | flagellar assembly protein FliH | | flagellar FliJ protein | | flagellar protein FliO | | flagellar biosynthetic protein FliP | | methyl-accepting chemotaxis protein | | flagellar protein | | | 0.43 | | --- | | 0.29 | | 0.24 | | 0.36 | | 0.44 | | 0.56 | | 0.60 | | 0.59 | |
| Metabolism genes | | *accA* | | --- | | *aceF* | | *ackA* | | *alkA* | | *alkB* | | *alr* | | *ansP* | | *argS* | | *bcsC* | | *cadA* | | *cafA* | | *cbiE* | | *cdd* | | *coaA* | | *cpsG* | | *dacA* | | *dapE* | | *deoD* | | *dsdX* | | *entC* | | *entE* | | *eutE* | | *fdnG* | | *fdoG* | | *fdoH* | | *fdoI* | | *fdx* | | *fruK* | | *fucK* | | *garD* | | *gcd* | | *glpB* | | *glpD* | | *glpF* | | *glpX* | | *gltX* | | *gpsA* | | *ispD* | | *ispF* | | *lipA* | | *lldP* | | *lpdA* | | *mdoH* | | *melA* | | *menF* | | *murG* | | *mutM* | | *nemA* | | *nrdA* | | *nrdB* | | *nrdG* | | *nupC* | | *nupG* | | *pcm* | | *pduP* | | *pduV* | | *pepB* | | *pgpA* | | *pheS* | | *phsA* | | *plsB* | | *potB* | | *pta* | | *pykF* | | *pyrE* | | *queA* | | *rfaL* | | *rfbD* | | *rfbV* | | *rhaD* | | *rhlE* | | *rimI* | | *rimK* | | *rpsA* | | *rpsA* | | *rpsA* | | *rpsA* | | *rpsA* | | *rpsA* | | *sdaB* | | *serS* | | *speF* | | *speG* | | *spoT* | | *spoU* | | *sppA* | | *srmB* | | STM1791 | | STM1795 | | STM1860 | | STM2722 | | STM3136 | | *tdh* | | *thiI* | | *thiL* | | *trmA* | | *trmE* | | *ubiA* | | *uhpB* | | *yggW* | | *ygiY* | | *yhbZ* | | *fruA* | | *fruF* | | *ptsA* | | STM3255 | | | acetyl-coenzyme A carboxylase carboxyl transferase subunit alpha | | --- | | dihydrolipoamide acetyltransferase component (E2) of pyruvate dehydrogenase | | acetate kinase | | DNA-3-methyladenine glycosidase II | | AlkB protein | | alanine racemase | | L-asparagine permease | | arginyl-tRNA synthetase | | probable endoglucanase precursor | | lysine decarboxylase | | ribonuclease G | | precorrin-6Y C5,15-methyltransferase [decarboxylating] | | cytidine deaminase | | pantothenate kinase | | phosphomannomutase | | D-alanine carboxypeptidase | | succinyl-diaminopimelate desuccinylase | | purine nucleoside phosphorylase | | DsdX permease | | isochorismate synthase EntC | | 2,3-dihydroxybenzoate-AMP ligase | | putative aldehyde dehydrogenase | | formate dehydrogenase (pseudogene) | | formate dehydrogenase-O, major subunit | | formate dehydrogenase-O beta subunit | | formate dehydrogenase-O gamma subunit | | ferredoxin | | 1-phosphofructokinase | | L-fuculose kinase | | D-galactarate dehydratase | | glucose dehydrogenase | | anaerobic glycerol-3-phosphate dehydrogenase subunit B | | aerobic glycerol-3-phosphate dehydrogenase | | glycerol uptake facilitator protein | | putative glycerol metabolic protein | | glutamyl-tRNA synthetase | | glycerol-3-phosphate dehydrogenase | | 2-C-methyl-D-erythritol 4-phosphate cytidylyltransferase | | 2C-methyl-D-erythritol 2,4-cyclodiphosphate synthase | | lipoic acid synthetase | | L-lactate permease | | dihydrolipoamide dehydrogenase | | periplasmic glucans biosynthesis protein MdoH | | alpha-galactosidase | | isochorismate synthase | | UDP-N-acetylglucosamine | | formamidopyrimidine-DNA glycosylase | | N-ethylmaleimide reductase | | ribonucleoside-diphosphate reductase 1 alpha chain | | ribonucleoside-diphosphate reductase 1 beta chain | | anaerobic ribonucleoside-triphosphate reductase activating protein | | nucleoside permease NupC | | nucleoside permease | | L-isoaspartyl protein carboxyl methyltransferase type II | | putative CoA-dependent proprionaldehyde dehydrogenase | | putative propanediol utilization protein PduV | | peptidase B (leucyl aminopeptidase) | | phosphatidylglycerophosphatase A | | phenylalanyl-tRNA synthetase alpha chain | | thiosulfate reductase precursor | | glycerol-3-phosphate acyltransferase | | spermidine/putrescine transport system permease protein PotB | | phosphate acetyltransferase | | pyruvate kinase | | orotate phosphoribosyltransferase | | S-adenosylmethionine:tRNA ribosyltransferase- isomerase | | O-antigen ligase | | dTDP-4-dehydrorhamnose reductase | | LPS side chain defect: abequosyltransferase | | rhamnulose-1-phosphate aldolase (pseudogene) | | putative ATP-dependent RNA helicase rhlE | | ribosomal-protein-alanine acetyltransferase | | ribosomal protein S6 modification protein | | 30S ribosomal protein S1 | | 30S ribosomal protein S1 | | 30S ribosomal protein S1 | | 30S ribosomal protein S1 | | 30S ribosomal protein S1 | | 30S ribosomal protein S1 | | L-serine dehydratase 2 (L-serine deaminase 2) | | anaerobic dimethyl sulfoxide reductase chain A precursor | | ornithine decarboxylase, inducible (pseudogene) | | spermidine N1-acetyltransferase | | guanosine-3',5'-bis(diphosphate) 3'- pyrophosphohydrolase; CG Site No. 156 | | tRNA (guanosine-2'-O)-methyltransferase | | protease IV | | ATP-dependent RNA helicase SrmB | | hydrogenase-1 operon protein HyaF | | glutamate dehydrogenase | | transposase (pseudogene) | | terminase, ATPase subunit | | D-mannonate oxidoreductase | | threonine 3-dehydrogenase | | thiamine biosynthesis protein ThiI | | thiamine-monophosphate kinase | | tRNA (uracil-5)-methyltransferase | | thiophene and furan oxidation protein | | 4-hydroxybenzoate octaprenyl transferase | | two-component system sensor histidine kinase | | possible oxygen-independent coproporphyrinogen III oxidase | | probable two-component system sensor histidine kinase | | probable GTP-binding protein | | PTS system, fructose-specific IIBC component | | pts system, fructose-specific IIA/FPR component | | General PTS family, enzyme I | | PTS system, sugar phosphotransferase enzyme IIBC component | | | 0.58 | | --- | | 0.27 | | 0.47 | | 0.42 | | 0.45 | | 0.64 | | 0.42 | | 0.52 | | 0.54 | | 0.31 | | 0.38 | | 0.61 | | 0.28 | | 0.48 | | 0.55 | | 0.51 | | 0.46 | | 0.26 | | 0.59 | | 0.46 | | 0.40 | | 0.66 | | 0.18 | | 0.10 | | 0.07 | | 0.08 | | 0.50 | | 0.24 | | 0.71 | | 0.43 | | 0.45 | | 0.06 | | 0.22 | | 0.47 | | 0.53 | | 0.60 | | 0.41 | | 0.39 | | 0.46 | | 0.67 | | 0.62 | | 0.39 | | 0.54 | | 0.13 | | 0.54 | | 0.76 | | 0.71 | | 0.56 | | 0.43 | | 0.42 | | 0.65 | | 0.62 | | 0.25 | | 0.55 | | 0.48 | | 0.44 | | 0.55 | | 0.46 | | 0.61 | | 0.35 | | 0.53 | | 0.39 | | 0.36 | | 0.48 | | 0.49 | | 0.32 | | 0.55 | | 0.60 | | 0.51 | | 0.49 | | 0.35 | | 0.47 | | 0.74 | | 0.50 | | 0.46 | | 0.44 | | 0.40 | | 0.33 | | 0.28 | | 0.07 | | 0.63 | | 0.30 | | 0.35 | | 0.39 | | 0.41 | | 0.53 | | 0.31 | | 0.55 | | 0.45 | | 0.62 | | 0.72 | | 0.48 | | 0.48 | | 0.41 | | 0.60 | | 0.56 | | 0.48 | | 0.49 | | 0.68 | | 0.57 | | 0.61 | | 0.42 | | 0.18 | | 0.15 | | 0.54 | | 0.43 | |
| **Membrane Proteins** | | *ompS* | | --- | | *yabI* | | | outer membrane protein S1 | | --- | | DedA family integral membrane protein | | | 0.68 | | --- | | 0.40 | |
| **Transport genes** | | *cadB* | | --- | | *caiT* | | *emrA* | | *emrB* | | *emrD* | | *fhuB* | | *fhuC* | | *gntU* | | *kdpB* | | *kup* | | *nhaB* | | *phnS* | | *proW* | | *pstC* | | *sapA* | | *sdaC* | | *setB* | | STM0057 | | STM3022 | | STM3134 | | *uhpT* | | *ybhF* | | *ychM* | | *proP* | | STM0818 | | *proX* | | | probable cadaverine/lysine antiporter | | --- | | probable carnitine transporter | | multidrug resistance protein A | | multidrug resistance protein B | | multidrug resistance protein D | | ferrichrome transport protein FhuB precursor | | ferrichrome transport ATP-binding protein FhuC | | low-affinity gluconate transporter | | potassium-transporting ATPase B chain | | membrane transport protein | | regulator of intracellular pH; Na+/H+ antiporter | | probable periplasmic binding component of 2- aminoethylphosphonate transporter | | glycine betaine/L-proline transport system permease protein P | | phosphate transport system permease protein | | peptide transport periplasmic protein SapA precursor | | putative serine transporter | | sugar efflux transporter | | citrate-sodium symporter | | probable amino acid transport protein | | hexuronate transporter | | hexosephosphate transport protein | | hypothetical ABC transporter ATP-binding protein | | putative sulphate transporter | | ProP - proline transport | | HlyD-family secretion protein | | glycine betaine-binding periplasmic protein precursor - proline transport | | | 0.27 | | --- | | 0.27 | | 0.36 | | 0.41 | | 0.44 | | 0.40 | | 0.38 | | 0.19 | | 0.48 | | 0.49 | | 0.62 | | 0.50 | | 0.20 | | 0.65 | | 0.69 | | 0.04 | | 0.42 | | 0.30 | | 0.22 | | 0.50 | | 0.64 | | 0.49 | | 0.68 | | 0.68 | | 0.44 | | 0.24 | |
| **Regulatory genes** | | sdiA | | --- | | STM3602 | | | cell-division regulatory protein | | --- | | gntR family regulatory protein | | | 0.51 | | --- | | 0.62 | |
| **Plas**mid Genes | | finP | | --- | | psiB | | PSLT059 | | PSLT062 | | traJ | | | putative transglycosylase | | --- | | Plasmid SOS inhibition | | putative adenine-specific DNA methylase | | putative cytoplasmic protein | | conjugative transfer: regulation | | | 0.54 | | --- | | 0.70 | | 0.64 | | 0.38 | | 0.64 | |
| **Phage genes** | | STM0903 | | --- | | STM0906 | | STM0915 | | STM0925 | | STM1030 | | STM2604 | | STM2703 | | | Fels-1 prophage; putative chaparone | | --- | | Fels-1 prophage | | Fels-1 prophage | | Fels-1 prophage; putative host specificity protein | | Gifsy-2 prophage | | Gifsy-1 prophage: similar to head protein gpshp of phage 21 | | Fels-2 prophage: similar to invertase (pin) in phage E14 | | | 0.56 | | --- | | 0.54 | | 0.47 | | 0.15 | | 0.70 | | 0.31 | | 0.69 | |
| **DNA and transcription related genes** | | cytR | | --- | | dnaB | | ecnR | | hepA | | hrpB | | lysR | | recO | | STM3124 | | topB | | uxuR | | eutR | | | transcriptional repressor | | --- | | replicative DNA helicase | | transcriptional regulatory protein | | probable ATP-dependent helicase HepA | | ATP-dependent helicase HrpB | | transcriptional activator protein LysR | | DNA repair protein RecO | | LysR-family transcriptional regulator | | DNA topoisomerase III | | uxu operon transcriptional regulator | | ethanolamine operon transcriptional regulator | |  | | | 0.56 | | --- | | 0.48 | | 0.51 | | 0.42 | | 0.57 | | 0.62 | | 0.50 | | 0.53 | | 0.66 | | 0.63 | | 0.46 | |
| Genes with unknown, miscellaneous or putative function | | STM3121 | | --- | | *yadQ* | | *yjfM* | | *slp* | | *sirC* | | *dinF* | | *yegO* | | *nusB* | | *ybfM* | | *yciA* | | *hcaT* | | *yajI* | | STM3098 | | *ydhM* | | *ygjP* | | *yaeJ* | | STM1527 | | *ydcN* | | *ydgO* | | *yaeB* | | STM0576 | | *yhjD* | | *pduM* | | STM3785 | | *yigQ* | | STM2747 | | STM0691 | | *yhiR* | | *yjdL* | | STM4493 | | STM3834 | | *yegH* | | *yeeF* | | STM3792 | | *yhjJ* | | *prfC* | | STM4433 | | *yidQ* | | *ygbJ* | | *yieO* | | STM1698 | | *yihR* | | *phnA* | | STM4186 | | STM0577 | | *recF* | | *yjiW* | | *yciU* | | STM0100 | | *yfgE* | | STM1256 | | STM1188 | | *stfG* | | *ampG* | | *ybhR* | | *stjB* | | STM0233 | | *yqjC* | | *yhjU* | | *fxsA* | | *yhjN* | | *yciH* | | STM3745 | | STM2135 | | STM4011 | | STM1128 | | *stfA* | | *sgaU* | | *yjdE* | | STM4065 | | *yiiP* | | *yifZ* | | STM3781 | | STM4216 | | STM0765 | | *stdC* | | *yccW* | | STM3141 | | *ydeW* | | *yigG* | | *eco* | | STM4012 | | *ygfE* | | *yneG* | | *flk* | | *yhgF* | | *yejM* | | STM0810 | | STM4309 | | STM4492 | | *yiiG* | | STM4498 | | *yebB* | | *fdnG* | | *mgtC* | | *orf48* | | *hscB* | | *yhfK* | | *yhhK* | | *rfaQ* | | STM3026 | | *ycfH* | | STM4010 | | *yojI* | | *wzxE* | | *yfhJ* | | STM2757 | | *yqiE* | | STM4496 | | STM1490 | | STM2234 | | *yfbQ* | | STM4417 | | STM1540 | | *res* | | *ynfA* | | *gidB* | | *yqjE* | | *pitA* | | *yqjK* | | *ygbQ* | | STM2767 | | STM1025 | | *yidC* | | STM3859 | | *yigC* | | *ydgR* | | STM4066 | | STM0274 | | STM4204 | | *yfhK* | | *gidA* | | STM2779 | | *cspA* | | *ygbO* | | STM2281 | | STM3631 | | *ybdG* | | *yhiP* | | STM3793 | | *yadB* | | *yhjV* | | *yggH* | | *prfB* | | STM1987 | | *yhfC* | | *ydeZ* | | *fdhE* | | *yifB* | | *fucU* | | *gntK* | | *nrfG* | | *pefI* | | STM0268 | | *ytfE* | | *adiY* | | STM0650 | | *yhjO* | | *orf5* | | *ydhC* | | *yaaH* | | *yjiY* | | | putative transcriptional regulator, LysR family | | --- | | putative membrane protein | | conserved hypothetical protein | | putative lipoprotein | | putative regulator | | putative DNA-damage-inducible membrane protein | | putative RND-family transporter protein | | N utilization substance protein B | | putative outer membrane protein | | putative acyl-coA hydrolase | | putative 3-phenylpropionate permease | | putative lipoprotein | | probable global regulatory protein | | putative TetR-family transcriptional regulator | | conserved hypothetical protein | | putative release factor | | putative membrane protein | | putative DNA-binding protein | | putative membrane protein | | conserved hypothetical protein | | putative transport protein, PTS system | | putative membrane protein | | conserved hypothetical protein | | putative GntR-family transcriptional regulator | | putative exported protein | | putative cytoplasmic protein | | conserved hypothetical protein | | conserved hypothetical protein | | putative transmembrane transport protein | | putative cytoplasmic protein | | putative LysR-family transcriptioanl regulator | | putative membrane protein | | putative amino acid transporter protein | | probable permease | | putative zinc-protease precursor | | peptide chain release factor 3 | | putative thiamine pyrophosphate-requiring enzyme, oxidoreductase | | putative lipoprotein | | putative oxidoreductase (pseudogene) | | putative transmembrane efflux protein (pseudogene) | | hypothetical protein | | conserved hypothetical protein | | conserved hypothetical protein | | putative cytoplasmic protein | | putative transport protein, PTS system | | recF protein | | conserved hypothetical protein | | conserved hypothetical protein | | hypothetical protein | | conserved hypothetical protein | | putative inner membrane transport protein | | putative inner membrane lipoprotein | | putative minor fimbrial subunit; putative adhesin | | AmpG protein | | putative inner membrane protein | | putative fimbrial usher protein | | putative secreted chitinase | | putative exported protein | | putative membrane protein | | FxsA protein | | putative polysaccharide biosynthesis protein subunit B | | putative translation initiaiton factor | | putative cytoplasmic protein | | hypothetical protein | | hypothetical protein | | putative membrane transporter | | putative fimbrial subunit | | conserved hypothetical protein | | putative amino acid permease | | putative membrane protein | | putative transmembrane efflux protein | | putative membrane protein | | putative carbohydrate kinase | | putative inner membrane protein | | putative cation transporter | | probable fimbrial chaperone protein | | conserved hypothetical protein | | hypothetical protein | | putative regulatory protein | | conserved hypothetical protein | | ecotin precursor | | hypothetical protein (pseudogene) | | conserved hypothetical protein | | conserved hypothetical protein | | Div protein | | putative transcription accessory protein | | putative sulphatase | | putative inner membrane protein | | putative exported protein | | putative cytoplasmic protein | | putative lipoprotein | | putative inner membrane protein | | conserved hypothetical protein | | fdnG? | | conserved hyopthetical protein | | putative amino acid permease | | chaperone protein HscB | | conserved hypothetical protein (pseudogene) | | conserved hypothetical protein | | lipopolysaccharide core biosynthesis protein | | hypothetical protein | | conserved hypothetical protein | | conserved hypothetical protein | | putative ABC transporter ATP-binding protein | | putative lipopolysaccharide biosynthesis protein | | conserved hypothetical protein | | putative cytoplasmic protein | | conserved hypothetical protein | | putative ATPase involved in DNA repair | | putative voltage gated chloride channel protein | | putative tail fiber assembly protein | | putative aminotransferase | | putative transcriptional regulator | | putative secreted hydrolase | | type III Restriction-modification system StyLTI enzyme res | | putative membrane protein | | glucose inhibited division protein | | putative membrane protein | | universal stress protein B | | conserved hypothetical protein | | conserved hypothetical protein | | putative Superfamily I DNA and RNA helicase | | putative lipoprotein | | putative membrane protein | | putative shikimate 5-dehydrogenase | | putative membrane protein | | putative proton/oligopeptide symporter | | putative carbohydrate kinase | | conserved hypothetical protein (pseudogene) | | putative inner membrane protein | | putative sensor kinase protein | | glucose inhibited division protein | | hypothetical protein | | cold shock protein | | conserved hypothetical protein | | putative transcriptional regulator | | putative permease (pseudogene) | | putative membrane protein | | putative PTR2 family transport protein | | putative carbohydrate kinase | | glutamyl-tRNA synthetase-related protein | | putative amino acid permease | | conserved hypothetical protein | | peptide chain release factor 2 (RF-2) | | putative membrane protein | | putative membrane protein | | putative ABC transporter, membrane component | | FdhE protein | | conserved hypothetical protein (pseudogene) | | fuscose operon fucU protein | | putative gluconokinase | | NrfG protein | | probable regulatory protein | | hypothetical protein | | conserved hypothetical protein | | putative AraC family regulatory protein | | putative hydrolase C-terminus | | putative polysaccharide biosynthesis protein catalytic subunit | | none | | putative integral membrane transport protein | | conserved hypothetical protein | | probable carbon starvation protein | | | 0.74 | | --- | | 0.72 | | 0.70 | | 0.69 | | 0.69 | | 0.69 | | 0.69 | | 0.69 | | 0.69 | | 0.68 | | 0.68 | | 0.68 | | 0.68 | | 0.68 | | 0.67 | | 0.67 | | 0.66 | | 0.66 | | 0.66 | | 0.66 | | 0.65 | | 0.65 | | 0.65 | | 0.65 | | 0.64 | | 0.64 | | 0.64 | | 0.64 | | 0.63 | | 0.63 | | 0.62 | | 0.62 | | 0.62 | | 0.62 | | 0.62 | | 0.61 | | 0.61 | | 0.61 | | 0.60 | | 0.60 | | 0.60 | | 0.60 | | 0.60 | | 0.60 | | 0.60 | | 0.60 | | 0.59 | | 0.59 | | 0.59 | | 0.59 | | 0.59 | | 0.59 | | 0.59 | | 0.59 | | 0.58 | | 0.58 | | 0.57 | | 0.57 | | 0.57 | | 0.57 | | 0.57 | | 0.57 | | 0.57 | | 0.57 | | 0.56 | | 0.56 | | 0.56 | | 0.56 | | 0.55 | | 0.55 | | 0.55 | | 0.54 | | 0.54 | | 0.54 | | 0.54 | | 0.54 | | 0.54 | | 0.54 | | 0.54 | | 0.53 | | 0.53 | | 0.53 | | 0.53 | | 0.53 | | 0.52 | | 0.52 | | 0.52 | | 0.52 | | 0.52 | | 0.52 | | 0.52 | | 0.52 | | 0.51 | | 0.51 | | 0.51 | | 0.51 | | 0.51 | | 0.51 | | 0.50 | | 0.50 | | 0.50 | | 0.50 | | 0.50 | | 0.50 | | 0.50 | | 0.49 | | 0.49 | | 0.49 | | 0.49 | | 0.49 | | 0.49 | | 0.48 | | 0.48 | | 0.48 | | 0.48 | | 0.48 | | 0.47 | | 0.46 | | 0.46 | | 0.45 | | 0.44 | | 0.43 | | 0.43 | | 0.43 | | 0.42 | | 0.42 | | 0.42 | | 0.42 | | 0.41 | | 0.40 | | 0.39 | | 0.38 | | 0.38 | | 0.37 | | 0.37 | | 0.37 | | 0.37 | | 0.37 | | 0.36 | | 0.35 | | 0.35 | | 0.35 | | 0.34 | | 0.33 | | 0.32 | | 0.32 | | 0.29 | | 0.29 | | 0.28 | | 0.27 | | 0.26 | | 0.25 | | 0.24 | | 0.23 | | 0.19 | | 0.18 | | 0.17 | | 0.14 | | 0.14 | | 0.12 | | 0.12 | | 0.05 | |

Genes changes with B>0 were taken as significant
